# Phytohormone Crosstalk Under Elevated CO_2_: Linking Sugar Signaling, Stress Resilience, and Metabolic Quality in Tea

**DOI:** 10.3390/biom16101447

**Published:** 2026-10-04

**Authors:** Abdul Wakeel Umar, Muhammad Naeem, Naveed Ahmad, Jiaen Zhang, Ming Xu

**Affiliations:** 1 Guangdong-Hong Kong Joint Laboratory for Carbon Neutrality, Jiangmen Laboratory of Carbon Science and Technology, Jiangmen 529199, China; umar@hkustgz-jcl.ac.cn (A.W.U.); naeembbt@gmail.com (M.N.); 2 Department of Ecology, College of Natural Resources and Environment, South China Agricultural University, Guangzhou 510642, China; 3 Institute for Safflower Industry Research of Shihezi University, Pharmacy College of Shihezi University, Key Laboratory of Xinjiang Phytomedicine Resource and Utilization, Ministry of Education, Shihezi 832003, China; naveed@shzu.edu.cn

**Keywords:** *Camellia sinensis*, elevated CO_2_, phytohormone crosstalk, sugar signaling, source–sink balance, photosynthetic acclimation, metabolic quality

## Abstract

Elevated atmospheric CO_2_ (eCO_2_) is commonly treated as a photosynthetic substrate, yet in horticultural crops it also alters carbon status and signaling processes associated with stress resilience and quality-related metabolism. Tea (*Camellia sinensis*) is particularly valuable because young-shoot metabolites are closely related to carbon–nitrogen balance and defense. This review develops an evidence-based framework linking guard-cell CO_2_ sensing and photosynthetic carbon influx with soluble-sugar and starch pools and candidate signaling through hexokinase (HXK), trehalose-6-phosphate (T6P), SNF1-related protein kinase 1 (SnRK1), and target of rapamycin (TOR). These crosstalk pionts intersect with phytohormone networks: abscisic acid (ABA)–Ca^2+^–reactive oxygen species (ROS)–nitric oxide (NO) signaling contributes to stomatal and water-stress responses; salicylic acid (SA)–NO promotes eCO_2_-induced flavonoid biosynthesis; jasmonic acid (JA)–ethylene contributes to activate defense and volatile metabolism; and auxin–ethylene influences growth and defense. Nitrogen availability and sink strength may determine whether additional carbon supports assimilation and quality metabolism or promotes carbohydrate feedback and photosynthetic acclimation. We relate these pathways to catechins, theanine, caffeine, and aroma volatiles while distinguishing tea-supported relationships from mechanisms derived mainly from model plants. Finally, we propose cumulative CO_2_ enrichment above baseline (Δppm·h) as a treatment-reporting descriptor and hypothesis-generating variable integrating enrichment magnitude and duration. Its biological relevance should be tested across nitrogen supply, genotype, developmental stage, and stress context using time-based gas exchange, stomatal phenotyping, hormone profiling, carbon–nitrogen flux analysis, and multi-omics. This model presents sugar–phytohormone crosstalk as a candidate regulatory junction linking carbon enrichment with stress responses and quality-related metabolism in tea plants.

## 1. Introduction

Tea (*Camellia sinensis* (L.) O. Kuntze) is a long-lived, evergreen, woody beverage crop of major global cultural and economic importance, with cultivation distributed across many countries and production systems [1,2]. Unlike commonly grown crops whose agronomic value is often judged primarily by how much biomass they produce, the tea plant is principally valued because harvested young leaves and buds are processed into various tea products whose market identity and consumer acceptance depend on sensory quality traits (taste, aroma, color, mouthfeel) that are influenced by the biochemical composition of the leaves [3,4]. Therefore, quality-related chemical composition in tea is commonly characterized by the concentrations and ratios of key biochemical constituents, including polyphenols (especially catechins), free amino acids (particularly L-theanine), alkaloids (such as caffeine), soluble sugars, and aroma-active volatiles, whose variation is known to differentiate product types and quality grades [3,5,6]. However, changes in individual metabolites or metabolite classes should not by themselves be interpreted as improvements in overall tea quality, because processed-tea chemistry, sensory attributes, and consumer acceptance represent distinct outcomes.

For example, polyphenols, particularly catechin-class flavonoids, are major contributors to tea infusion color and flavor, while free amino acids enhance freshness and aroma, and soluble sugars contribute to sweetness [5,7]. High-performance liquid chromatography (HPLC)-based analysis, using retention times and photodiode-array ultraviolet (PDA-UV) spectra for compound identification and external-standard calibration for quantification, showed marked variation in theanine, caffeine, and individual catechins across different plucking periods [5]. Such compositional variation provides information on quality-related chemistry but does not, without sensory or processed-product evaluation, demonstrate superior tea quality.

Tea quality chemistry is driven by metabolites that are influenced by carbon (C) assimilation, nitrogen (N) acquisition, and specialized metabolism. Catechins and flavonoids are emphasized as tea-specific compounds important for quality and for plant defense, linking sensory traits to ecological function [8,9]. These roles should be distinguished: altered flavonoid abundance does not necessarily indicate improved sensory quality or greater resistance to a particular biotic or abiotic stress. Transcriptomic datasets have been developed to map candidate genes for these pathways, indicating that the accumulation of catechins and related flavonoids is regulated rather than a passive outcome of growth [8,10]. Likewise, caffeine biosynthesis has been characterized as developmentally controlled, concentrated in young leaves and buds, and decreasing with leaf maturation [11].

**Figure 1:**
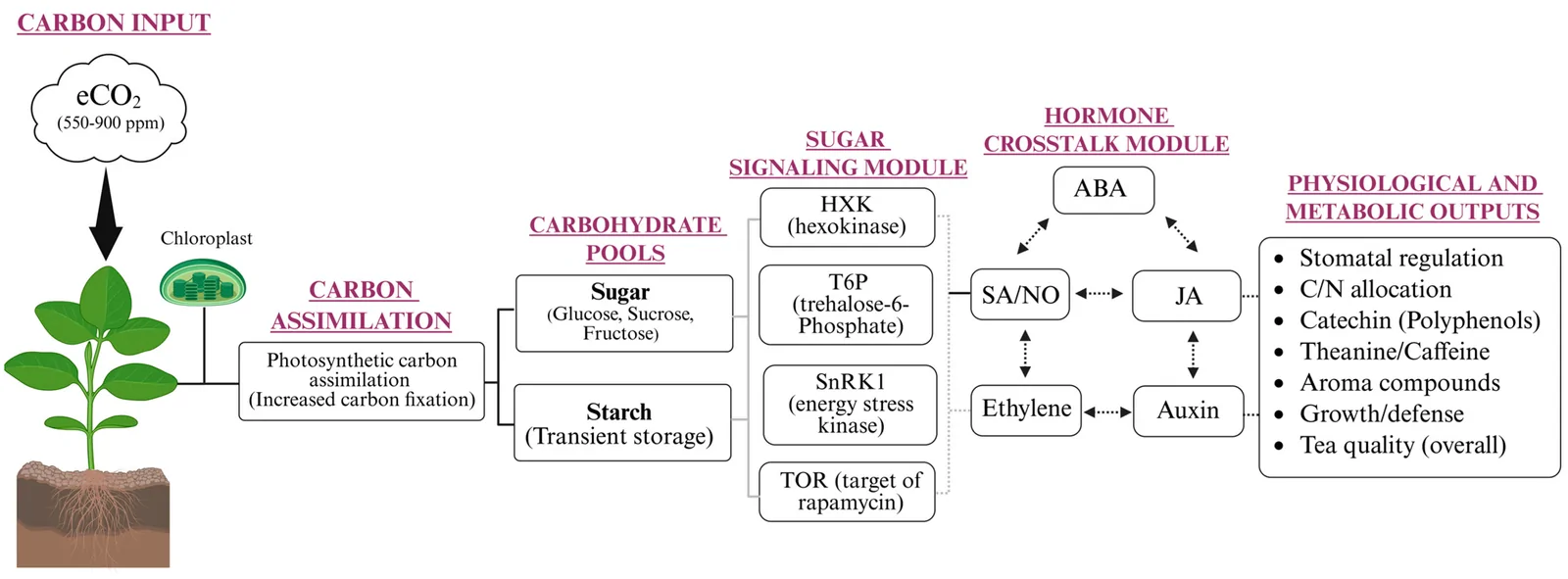
Systemic coordination of sugar signaling and phytohormone networks governing tea quality under elevated CO_2_ (eCO_2_). Elevated CO_2_ (550–900 ppm) increases carbon input and accelerates photosynthetic carbon assimilation, elevating soluble sugar (glucose, sucrose, fructose) and starch pools. These carbohydrate changes engage a central sugar-signaling module (HXK, T6P, SnRK1, and TOR). The sugar-signaling module is proposed to interact with a multilayered phytohormone network (ABA, SA/NO, JA, ethylene, and auxin). This integration coordinates downstream physiological outputs, including stomatal regulation and C/N allocation, while modulating secondary metabolism (catechins/polyphenols, theanine, caffeine, and aroma compounds) that collectively influence tea plant growth, defense, and overall quality. Solid black arrows indicate relationships directly supported by experimental evidence in tea plants (*Camellia sinensis*) under elevated CO_2_. Solid grey arrows represent conserved mechanisms primarily supported by evidence from other plant species. Dotted arrows denote hypothetical or model-based interactions. Figure created with BioRender.com.

A critical nitrogen-related component of tea quality is L-theanine, a non-protein amino acid that significantly contributes to the umami flavor of green tea and is commercially valuable outside tea beverages [12]. Genomic and biochemical studies indicate that theanine is synthesized from glutamate and ethylamine through the activity of theanine synthetase [12]. Physiological studies further show that theanine is formed predominantly in roots and transported to shoots, where it is closely integrated with nitrogen assimilation and carbon metabolism and may contribute to nitrogen storage, transport, and remobilization within the tea plant [10,13]. This integration is consistent with evidence that the accumulation of amino acids and flavonoids in young shoots correlates strongly with carbon and nitrogen metabolism across plant tissues, supporting a whole-plant coordination model for quality-related metabolite formation [13,14] (Figure 1).

Caffeine biosynthesis is most active in young leaves and buds, providing a rationale for why plucking standards and shoot developmental stage are central to the chemical profile of tea [10,11]. Metabolite profiling across plucking periods shows that concentrations of key constituents (including catechins, caffeine, and theanine) vary with harvest timing, linking seasonal/developmental dynamics to commercial quality [5,9]. Because these compounds derive from photosynthate and interact with nitrogen metabolism (e.g., theanine as a nitrogen reservoir), tea quality is sensitive to shifts in carbon availability and distribution across tissues [13].

Understanding CO_2_ effects on tea requires careful differentiation between direct carbon fertilization responses and indirect climate-change drivers (e.g., altered precipitation, drought, temperature extremes, and compound stresses) that also influence tea plant growth and chemistry [9,15]. Transcriptomic analyses suggest that drought can decrease quality and induce changes in metabolites linked to quality, reinforcing that water stress can confound results if CO_2_ is not controlled experimentally [9,15]. Similarly, combined cold-drought stress has been shown to cause complex responses impacting both growth and metabolite systems related to tea quality [2,16].

Research on eCO_2_ in tea plants has documented changes in photosynthetic carbon assimilation, primary and secondary metabolism, and quality-related compounds, while broader evidence also links eCO_2_ responses to water relations, defense, and environmental adaptation [2,17]. However, the mechanistic evidence is variable. Experimental studies in tea plants provide comparatively strong support for physiological and metabolic responses and for SA–NO involvement in eCO_2_-induced flavonoid biosynthesis, whereas the proposed roles of ABA, jasmonic acid, ethylene, auxin, and the HXK–T6P–SnRK1–TOR energy-signaling network are supported mainly by evidence from model plants or other crops [18,19,20]. Accordingly, this review distinguishes among three evidence levels: mechanisms demonstrated directly in tea plants under eCO_2_, conserved mechanisms supported in other plant systems, and testable hypotheses that remain to be validated in tea plants. This distinction is essential to prevent a theoretical mechanism from being presented as tea plant-specific experimental evidence.

Against this evidence-graded background, we consider eCO_2_ not simply as a photosynthetic substrate but as an environmental signal that alters cellular carbon status and interacts with phytohormone networks governing stress resilience and metabolic quality. This perspective is particularly relevant to the tea plant, a perennial horticultural crop in which repeated harvesting, developmental source–sink relationships, nitrogen availability, and stress-responsive secondary metabolism determine how additional carbon is partitioned among growth, storage, defense, and the quality-related composition of harvested shoots [21,22]. We integrate photosynthetic carbon entry and sugar sensing with abscisic acid (ABA)-centered stomatal regulation, salicylic acid (SA)–nitric oxide (NO)-mediated flavonoid defense, jasmonic acid (JA)–ethylene-associated defense and volatile metabolism, and auxin–ethylene regulation of growth–defense allocation, and auxin–ethylene regulation of growth–defense allocation [18,20,23,24,25]. We further connect these pathways to stomatal plasticity, carbon–nitrogen coordination, and the accumulation of catechins, theanine, caffeine, and aroma volatiles. Finally, we propose a testable dose- and context-dependent framework for determining when CO_2_ enrichment can strengthen stress adaptation and metabolic quality, while identifying the hormone-related mechanisms that require direct validation in tea plants.

### Literature Identification and Evidence Assessment

This article is a targeted critical review rather than a registered systematic review. To improve transparency and reproducibility, the literature relevant to eCO_2_ responses in tea plants was identified through structured searches of major bibliographic databases, supplemented by backward- and forward-citation tracing. Tea plant-specific searches combined “*Camellia sinensis*” or “tea plant” with terms related to eCO_2_, CO_2_ enrichment, photosynthesis, stomatal regulation, source–sink relations, carbon–nitrogen coordination, sugar signaling, phytohormones, stress responses, and quality-related metabolites. Mechanism-specific searches were subsequently used to identify primary studies from model plants or other species where corresponding molecular relationships had not been examined directly in tea plants. Recent literature was prioritized when it updated mechanistic understanding, while older studies were retained when they provided foundational or uniquely relevant primary evidence.

Primary tea plant studies were prioritized when they imposed an identifiable eCO_2_ treatment and reported physiological, biochemical, molecular, defense-related, or quality-associated endpoints. Tea plant studies performed under other environmental or experimental conditions were retained when they provided species-specific evidence relevant to a proposed mechanism. Model-plant and cross-species studies were included when they supplied mechanistic evidence for signaling relationships that remain unresolved in tea plants. Review articles were used principally to identify recent developments, synthesize broader mechanisms, and trace relevant primary studies rather than as substitutes for primary evidence where causal relationships were discussed. Studies involving combined treatments were interpreted as evidence for an isolated CO_2_ effect only when the experimental design permitted the CO_2_ effect to be separated from the accompanying factor or interaction.

Evidence was evaluated along two complementary dimensions. First, the experimental context of each relationship was distinguished as: direct evidence from tea plants exposed to eCO_2_; tea plant-specific evidence generated outside the eCO_2_ context; mechanistic evidence from model plants or other species; or a relationship not yet tested directly in tea plants. Second, the strength of causal support was evaluated as descriptive or correlative, temporally or physiologically associative, pharmacologically perturbation-supported, or genetically/biochemically supported, where applicable. A change in hormone, metabolite, transcript, or physiological trait abundance alone was therefore not considered evidence that the measured factor mediates the eCO_2_ response. This two-dimensional assessment was used to define the maximum inference justified for each relationship and to avoid interpreting mechanistic evidence from other species as tea plant-specific causal evidence. Table 1 summarizes the principal relationships according to experimental context and strength of causal support, and Supplementary Table S1 provides the corresponding study-level experimental conditions, approaches, evidential limitations, and maximum inference supported by each study [17,18,23,24,25,26,27,28,29,30,31,32,33,34,35,36,37]. Together, these tables distinguish direct eCO_2_ responses in tea plants from tea plant-context mechanisms, cross-species mechanistic evidence, and relationships that remain experimentally unresolved in *Camellia sinensis*.

## 2. Elevated CO_2_ Reshapes Carbon Assimilation, Source–Sink Balance, and Carbon–Nitrogen Coordination

Tea plant-specific CO_2_ enrichment evidence shows that eCO_2_ can enhance net photosynthesis and shift carbon metabolism, increasing substrate availability for downstream pathways and contributing to altered primary and secondary metabolism linked to green tea quality [2,17]. Tea enrichment evidence suggests that enhanced photosynthesis under eCO_2_ can increase carbohydrate availability and energy demand, with downstream implications for respiration-related metabolism and allocation into anabolic pathways [17]. At the broader C_3_-plant level, the carbon response to eCO_2_ reflects coordinated changes in photosynthesis, photorespiration, and respiration, with its magnitude further conditioned by temperature, water, and nutrient availability [39]. This framing is consistent with general CO_2_ response theory emphasizing that the magnitude and persistence of photosynthetic stimulation depend on downstream utilization capacity (sink demand) and nutrient availability, particularly nitrogen [21,39,40,41]. This coordination also influences stress adaptation. When nitrogen supply and sink activity are sufficient, additional assimilated carbon can support growth, cellular repair, osmotic regulation, antioxidant metabolism, and the production of protective secondary metabolites. Conversely, when nitrogen or sink capacity is limited, carbon may accumulate as soluble sugars and starch without proportionally improving physiological resilience. Consequently, interpreting photosynthesis enhancement in tea plants requires joint consideration of carbon gain, carbon consumption, and metabolite partitioning into quality-relevant pools (amino acids and flavonoids) known to respond to nitrogen status and whole-plant C/N coordination [13,42,43].

Recent tea plant studies further show that nitrogen effects on quality-related metabolism are compound-, tissue-, and context-dependent. Nitrogen fertilization can differentially alter amino acids, theanine, caffeine, catechins, and aroma-related compounds, while nitrogen form and deficiency reshape theanine metabolism, and nitrate-responsive transport and assimilation pathways provide additional control points for N acquisition [6,44,45]. These responses therefore should not be interpreted as evidence that increasing N supply uniformly improves all quality-related traits, and their contribution to tea plant responses under eCO_2_ remains untested. Cross-crop evidence further indicates that greater carbon assimilation or biomass under eCO_2_ does not necessarily translate into improved nutrient composition, because dilution of nitrogen and mineral nutrients and interactions with genotype and environmental conditions can modify harvested-product composition [46]. Whether comparable dilution–allocation relationships occur in tea shoots requires direct testing.

Photosynthetic acclimation, involving partial downregulation of photosynthetic capacity under prolonged eCO_2_, can occur and is interpretable through A/ci responses and the emergence of downstream limitations, particularly nitrogen limitation and sink capacity constraints [21,47]. Methodological comparisons further indicate that photosynthetic acclimation may be accentuated under restricted rooting volumes, because constrained root growth and sink development can promote carbohydrate accumulation and enhance feedback limitations on photosynthesis [48,49].

**Figure 2:**
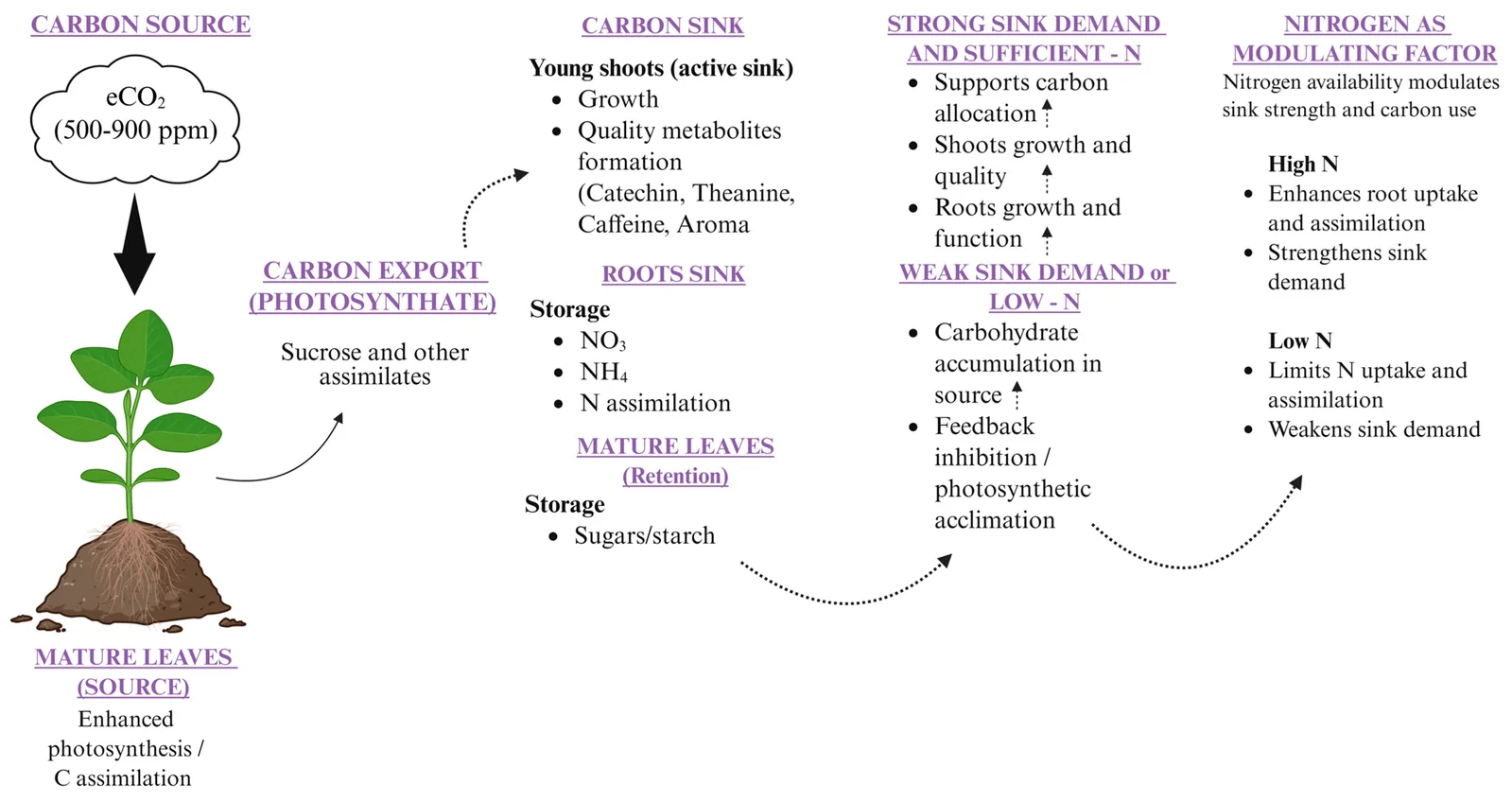
Crosstalk between source–sink regulation and nitrogen availability under elevated CO_2_ (eCO_2_) in tea. Photosynthetic carbon fixed by mature source leaves under eCO_2_ is converted into sucrose and other assimilates and exported to distinct carbon sinks (young shoots, roots, and mature-leaf storage pools). In young shoots (active sinks), this carbon influx supports growth and the accumulation of key quality metabolites (catechins, theanine, caffeine; aroma compounds remain less certain). High nitrogen availability strengthens root uptake/assimilation and overall sink demand, favouring carbon allocation to growth and quality. Under nitrogen limitation or weak sink demand, excess non-structural carbohydrates accumulate in source leaves, triggering feedback inhibition and photosynthetic acclimation. Nitrogen thus acts as a key modulating factor of sink strength and carbon-use efficiency. Solid black arrows indicate relationships directly supported by experimental evidence in tea plants (*Camellia sinensis*) under elevated CO_2_. Solid grey arrows represent conserved mechanisms primarily supported by evidence from other plant species. Dotted arrows denote hypothetical or model-based interactions. Figure created with BioRender.com.

These photosynthetic responses establish the biochemical precondition for downstream signaling processes. Once eCO_2_ increases carbon assimilation and carbohydrate availability, the central mechanistic question shifts from whether carbon supply increases to how plants perceive and regulate this altered carbon status. A critical component of this regulation is source–sink balance, which constrains the sustainability of photosynthetic stimulation under eCO_2_ (Figure 2). Across C_3_ species, initial increases in photosynthetic rates are frequently followed by partial acclimation, often associated with carbohydrate accumulation in source tissues and insufficient sink capacity to utilize the additional assimilates. When carbon acquisition exceeds the combined capacity of growth, storage, secondary metabolism, and nitrogen-supported protein synthesis, source leaves may become progressively sink-limited. This phenomenon, commonly described as sink limitation or feedback inhibition, has been extensively documented in both annual and perennial systems and is closely linked to the downregulation of photosynthetic gene expression and nitrogen reallocation [22,47,50]. In perennial crops such as tea, where growth is episodic and harvest targets young developing shoots, source–sink dynamics are particularly complex, involving interactions between mature source leaves, developing sinks, and management practices such as pruning and plucking. This acclimation is relevant to stress resilience because abundant leaf carbohydrates do not necessarily indicate greater functional resilience; sustained protection also requires nitrogen-dependent enzymes, proteins, and metabolic sinks capable of converting additional carbon into defense and repair processes.

Collectively, the effects of eCO_2_ on tea plants depend not only on the amount of carbon fixed but also on whether that carbon can be coordinated with nitrogen acquisition and directed toward active sinks. ECO_2_ may therefore support stress adaptation when nitrogen availability and sink demand sustain photosynthesis and the allocation of resources to growth, repair, and protective metabolism. When carbon supply exceeds utilization capacity, however, carbohydrate feedback and photosynthetic acclimation may reduce these benefits (Figure 2). This source–sink and carbon–nitrogen context provides the physiological foundation for the sugar–phytohormone signaling mechanisms discussed in the following section.

## 3. Sugar Signaling at the Carbon–Phytohormone Interface

**Figure 3:**
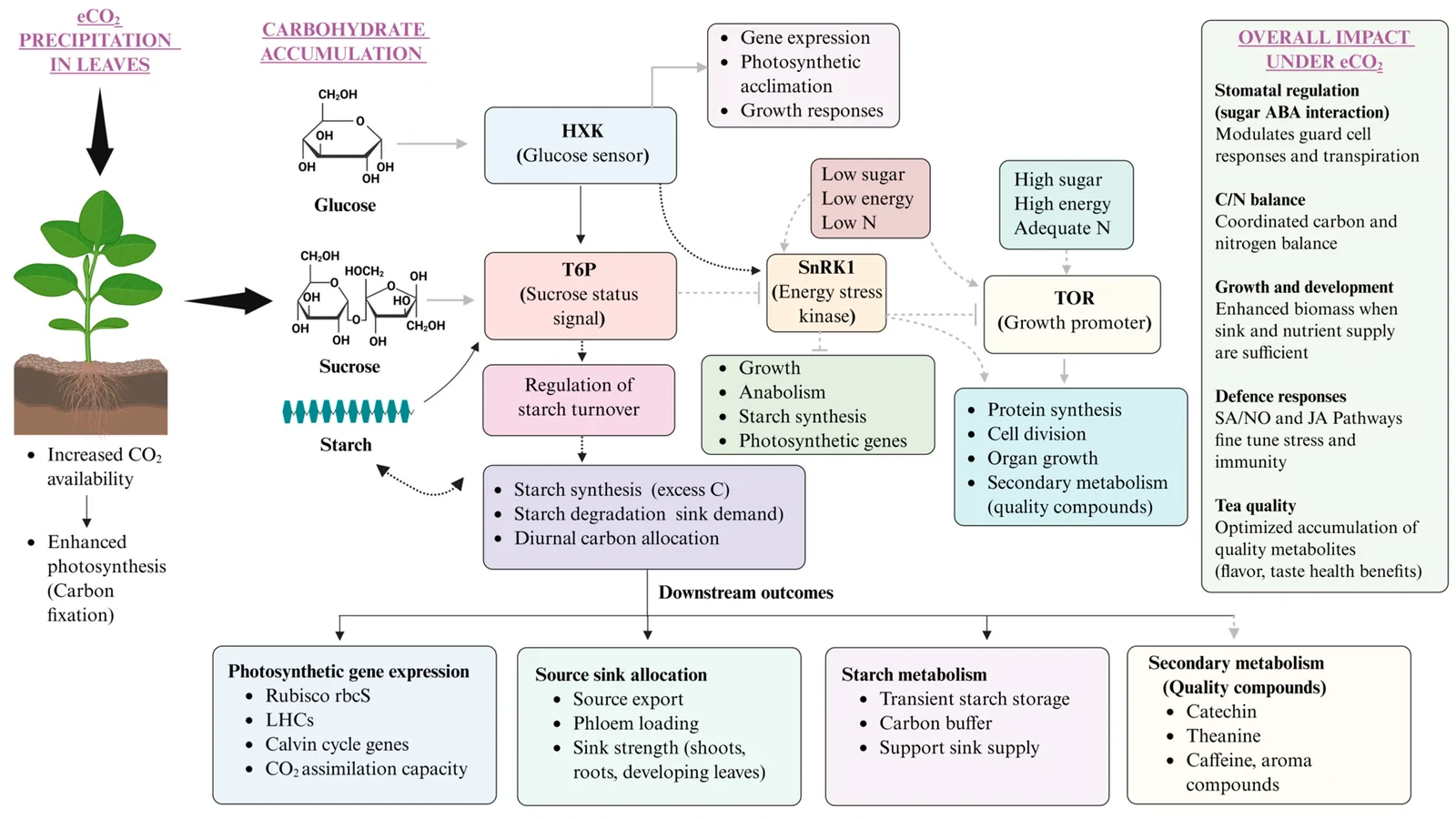
The sugar-signaling and regulatory network modulating tea quality under elevated CO_2_ (eCO_2_). Atmospheric CO_2_ enrichment enhances photosynthetic carbon fixation in *Camellia sinensis*, leading to the accumulation of glucose, sucrose, and starch. These carbohydrates function as metabolic signals rather than solely as storage compounds. Glucose is sensed by hexokinase (HXK), influencing gene expression, photosynthetic acclimation, and growth responses, while sucrose status is monitored via trehalose-6-phosphate (T6P). Together with energy and nitrogen cues, these signals coordinate the antagonistic activities of SnRK1 (energy-stress kinase) and TOR (growth promoter). This central regulatory core drives downstream outcomes, including source–sink allocation, starch turnover, photosynthetic gene expression, and the reprogramming of secondary metabolism that shapes key tea quality traits (catechins, theanine, caffeine; aroma compounds remain less certain). Solid black arrows indicate relationships directly supported by experimental evidence in tea plants (*Camellia sinensis*) under elevated CO_2_. Solid grey arrows represent conserved mechanisms primarily supported by evidence from other plant species. Dotted arrows denote hypothetical or model-based interactions. Figure created with BioRender.com.

Elevated CO_2_ affects plants not only by increasing the substrate availability for photosynthetic carboxylation (thereby raising carbon gain) but also by altering post-photosynthetic metabolism and regulatory processes that interpret carbohydrate status as information about resource availability [21]. Reviews of cellular responses to eCO_2_ emphasize that stimulation of photosynthesis is coupled to changes in post-photosynthetic processes that include carbon and nitrogen metabolism and hormonal regulation (Figure 3), framing carbohydrate accumulation as a likely upstream driver of broader metabolic reprogramming rather than a passive endpoint [51].

In tea plants, eCO_2_ has been associated with changes in physiological and defense-related responses, indicating that its effects should be interpreted not only in terms of increased carbon assimilation but also through regulatory pathways linking carbon status with defense, quality-related metabolism, and stress resilience [17,23,24,26]. Since tea quality is determined by specialized metabolites and amino acids whose biosynthesis depends on carbon and nitrogen allocation, tea plants provide a hypothetical system in which carbohydrate accumulation under eCO_2_ can act as a signal coordinating resource partitioning into quality-defining pathways [13,14,17]. Across C_3_ species, eCO_2_ increases net CO_2_ assimilation, and the resulting carbon influx tends to increase leaf carbohydrate pools (soluble sugars and transitory starch), especially when sinks are constrained by nutrients or developmental limits [52]. This evidence is consistent with broader syntheses emphasizing that carbon gain stimulation under eCO_2_ is linked to post-photosynthetic metabolic changes that encompass carbon metabolism and energy signaling [51].

Sugars regulate plant gene expression at a global scale, providing a mechanism for coordinating resource distribution among tissues and organs. Carbohydrate depletion upregulates genes for photosynthesis, remobilization, and export, while abundant sugars induce and repress distinct gene sets associated with storage and utilization [53]. This sugar-dependent transcriptional control provides a mechanistic explanation for how carbohydrate accumulation under eCO_2_ can alter leaf metabolism beyond mass-action effects, since sugar status directly modulates gene expression programs governing photosynthetic capacity, carbon partitioning, and sink–source coordination [53]. Reviews of sugar signaling in development emphasize that sugar signals operate throughout the plant life cycle, influencing developmental transitions and growth decisions as carbon availability changes [54].

Hexokinase (HXK) functions both as a glucose-phosphorylating enzyme and as a glucose-status sensor in several model plants, where it can influence photosynthetic gene expression, growth, and carbon allocation [18,35]. Under eCO_2_, increased soluble-sugar availability may therefore influence HXK-associated signaling and contribute to metabolic feedback [18] (Figure 3). However, eCO_2_-induced activation of an HXK-dependent pathway has not been demonstrated directly in tea plants. Its involvement in *Camellia sinensis* should consequently be regarded as a theoretical, testable mechanism rather than an established response.

Trehalose 6-phosphate (T6P) is widely regarded as an indicator and regulator of sucrose availability, whereas Snf1-related protein kinase 1 (SnRK1) and target of rapamycin (TOR) coordinate cellular responses to energy limitation and carbon abundance, respectively [36,37,52]. Evidence from model plants indicates that increased sucrose availability may elevate T6P in a tissue- and developmental-stage-dependent manner, potentially restricting SnRK1-associated starvation programs and favoring anabolic growth. Carbon and energy availability may also promote TOR-associated processes, including protein synthesis, cell proliferation, and sink development [18,52,55,56]. Nevertheless, T6P, SnRK1, and TOR should not be presented as a simple linear pathway, because their interactions are conditional and only partly resolved. Their activities have not been measured directly in tea plants exposed to eCO_2_; therefore, the proposed T6P–SnRK1–TOR response represents a cross-species mechanistic hypothesis requiring direct validation in tea plants. Current syntheses show that *SnRK1* and the target of rapamycin (*TOR*) pathway function as coordinated metabolic regulators, shaping development and growth by integrating signals of energy availability; *SnRK1* is associated with low-energy signaling while TOR relates to growth-promoting conditions [18,37,52]. Rather than being activated only under low-energy stress, *SnRK1* can also be repressed by high-energy signals, including sugar phosphates such as *T6P*, glucose-6-phosphate, and glucose-1-phosphate. This provides a mechanistic basis for interpreting eCO_2_-driven carbohydrate accumulation as a signal that shifts regulatory balance towards anabolic metabolism and storage, depending on sink and nutrient status [38,55,57].

*T6P* is recognized as a significant signaling metabolite involved in regulating plant growth and development in response to carbon availability (particularly sucrose), positioning it as a potential mediator through which eCO_2_-driven sucrose accumulation can be translated into developmental and metabolic reprogramming [38,58]. Mechanistically, *T6P* inhibits *SnRK1* activity and thus regulates metabolic pathways, providing a biochemical pathway through which sucrose/*T6P* can repress energy stress programs and promote growth/storage programs under high carbon availability [36,38]. Given that eCO_2_ is associated with increased carbohydrate availability and post-photosynthetic metabolic change, the *T6P*–*SnRK1* axis provides a mechanistic hypothesis for how eCO_2_-induced sugar accumulation can (i) increase starch accumulation, (ii) alter remobilization dynamics, and (iii) shift global metabolism and development. This includes tea plant-specific allocation toward metabolites defining quality versus storage, depending on sink demands [14,50,51].

Carbohydrate accumulation may contribute to feedback regulation of photosynthesis when carbon acquisition exceeds sink utilization [53]. In other plant systems, sugar-status signaling has been associated with altered expression of photosynthetic genes, reduced Rubisco abundance or activity, and the development of photosynthetic acclimation [50,59]. HXK-, T6P-, SnRK1-, and TOR-associated processes could participate in this response, but their causal involvement under eCO_2_ has not been established in tea plants. Tea plant-specific evidence currently supports changes in carbohydrate pools and photosynthetic performance more strongly than it supports the signaling pathway proposed to connect them. This interpretation is supported by broader analyses of CO_2_ responses and downstream limitations (especially nitrogen supply) when predicting the magnitude and longevity of CO_2_ stimulation, as nutrient supply limitations can restrict sink development and heighten the likelihood of carbohydrate feedback inhibition [21,40]. For tea plants, where nitrogen inputs crucially influence photosynthetic capacity and where quality-related metabolites represent substantial carbon and nitrogen pools, sugar-mediated feedback under eCO_2_ likely interacts with nitrogen availability to determine whether carbon is directed toward growth, starch storage, or quality-dependent secondary metabolism [17,42,43]. Within this evidence-graded framework, eCO_2_-induced changes in tea plant carbohydrate pools are proposed to alter cellular carbon status, which may subsequently be interpreted through HXK-, T6P-, SnRK1-, and TOR-associated signaling. These networks could intersect with phytohormone pathways regulating stomatal behavior, source–sink allocation, defense, and secondary metabolism [52,56]. However, tea plant experiments under eCO_2_ have not yet demonstrated activation of these signaling nodes or established the direction and timing of their interactions. The proposed network therefore integrates tea plant physiological evidence with conserved mechanisms identified in other plants and should be tested using time-resolved measurements of sugars, T6P, kinase activity, gene expression, hormones, and metabolic outputs (Figure 3).

Despite the strength of this conceptual framework, direct evidence in tea plants remains limited. Most mechanistic insights into *HXK*-, *T6P*-, *SnRK1*-, and *TOR*-mediated regulation derive from model species such as *Arabidopsis thaliana* and major crops, with relatively few studies quantifying these pathways in tea plants under carbon or CO_2_ change. Future work should therefore prioritize tea plant-specific investigations of sugar signaling, including the expression and activity of key sensing components (*HXK*, *TPS*/*TPP* genes, *SnRK1*, and *TOR*), as well as the identification of sugar-responsive transcriptional modules in developing tea shoots. Integrating such data with physiological and metabolomic measurements will be essential to validate whether the conserved carbon-signaling paradigms established in model systems fully explain CO_2_ responses in tea or whether crop-specific regulatory features emerge.

## 4. Phytohormone Crosstalk Governs Stress Resilience and Metabolic Quality Under Elevated CO_2_ in Tea Plants

Elevated CO_2_ may influence tea plant stress responses through interactions among cellular carbon status, phytohormone signaling, redox regulation, and secondary metabolism [2,60,61]. These interactions should not, however, be represented as a single established signaling cascade. The available evidence ranges from mechanisms demonstrated directly in tea plants under eCO_2_ to hormone functions observed under other environmental conditions and regulatory relationships inferred from model plants. Distinguishing these evidence levels is essential because changes in hormone concentration, stomatal conductance, or metabolite abundance do not by themselves establish a causal pathway linking CO_2_ perception to stress-responsive metabolism [2,60,62].

ABA provides a particularly important example of this distinction. Rapid stomatal closure in response to increased intercellular CO_2_ begins with guard-cell CO_2_/HCO_3_^−^ signaling and should not be equated with ABA perception [19,32,33,34,63]. Model-plant studies indicate that CO_2_- and ABA-responsive pathways may converge on Ca^2+^ dynamics, reactive oxygen species, nitric oxide, protein kinases, and anion-channel regulation, but convergence at shared downstream components does not make ABA the primary CO_2_ sensor [63,64,65,66,67]. In tea plants, exogenous ABA has altered drought-responsive proteins and carbon metabolism, while ABA and fluridone treatments support an ABA-associated contribution to high-light-induced anthocyanin accumulation [27,28]. These findings establish ABA functions in tea plants under drought and high-light conditions but do not demonstrate that eCO_2_ increases ABA or that ABA causes the reduction in stomatal conductance observed in well-watered tea plants [2]. ABA should therefore be presented as a potential integrator of CO_2_-modified water relations and environmental stress rather than as a demonstrated mediator of the tea plants’ eCO_2_ response.

The strongest tea plant-specific evidence concerns the SA–NO pathway. Tea plants exposed to eCO_2_ showed increased SA, NO, phenylalanine ammonia-lyase activity, and flavonoid accumulation [23]. Pharmacological suppression of SA biosynthesis or scavenging of NO reduced the eCO_2_-induced flavonoid response, while pathway-interference experiments placed SA predominantly upstream of NO [23]. These results provide direct evidence that SA–NO signaling hierarchy contributes to eCO_2_-induced flavonoid biosynthesis in tea plants. This pathway offers a mechanistic connection among carbon enrichment, hormone signaling, redox regulation, and the accumulation of quality- and defense-associated metabolites [8,9,23,43]. However, greater flavonoid accumulation should not automatically be interpreted as enhanced resilience across stress conditions, because resistance to a particular stressor depends on stress identity, defense costs, and interactions with other hormonal pathways [61,68].

JA responses under eCO_2_ appear more context-dependent. In two-year-old ‘Longjing 43’ seedlings, exposure to 800 μmol mol^−1^ CO_2_ for 14 days reduced foliar JA and was associated with decreased caffeine-mediated resistance to Colletotrichum gloeosporioides [24]. In contrast, another tea plant experiment using younger seedlings and longer CO_2_ exposure reported increased JA and SA concentrations [26]. Differences in genotype, plant age, exposure duration, nutritional conditions, and stress context may therefore alter both the direction and biological consequences of the JA response [24,26]. JA–ethylene crosstalk is widely involved in inducible defense and volatile metabolism in other plant systems [61,68]. Tea plant-context experiments indicate that ethylene signaling can modify catechin accumulation and antioxidant capacity [25]. Nevertheless, a causal JA–ethylene module connecting eCO_2_ with defense or aroma metabolism has not been demonstrated in tea plants. It should therefore be presented as a candidate regulatory interaction supported by cross-species and tea plant-context evidence.

**Figure 4:**
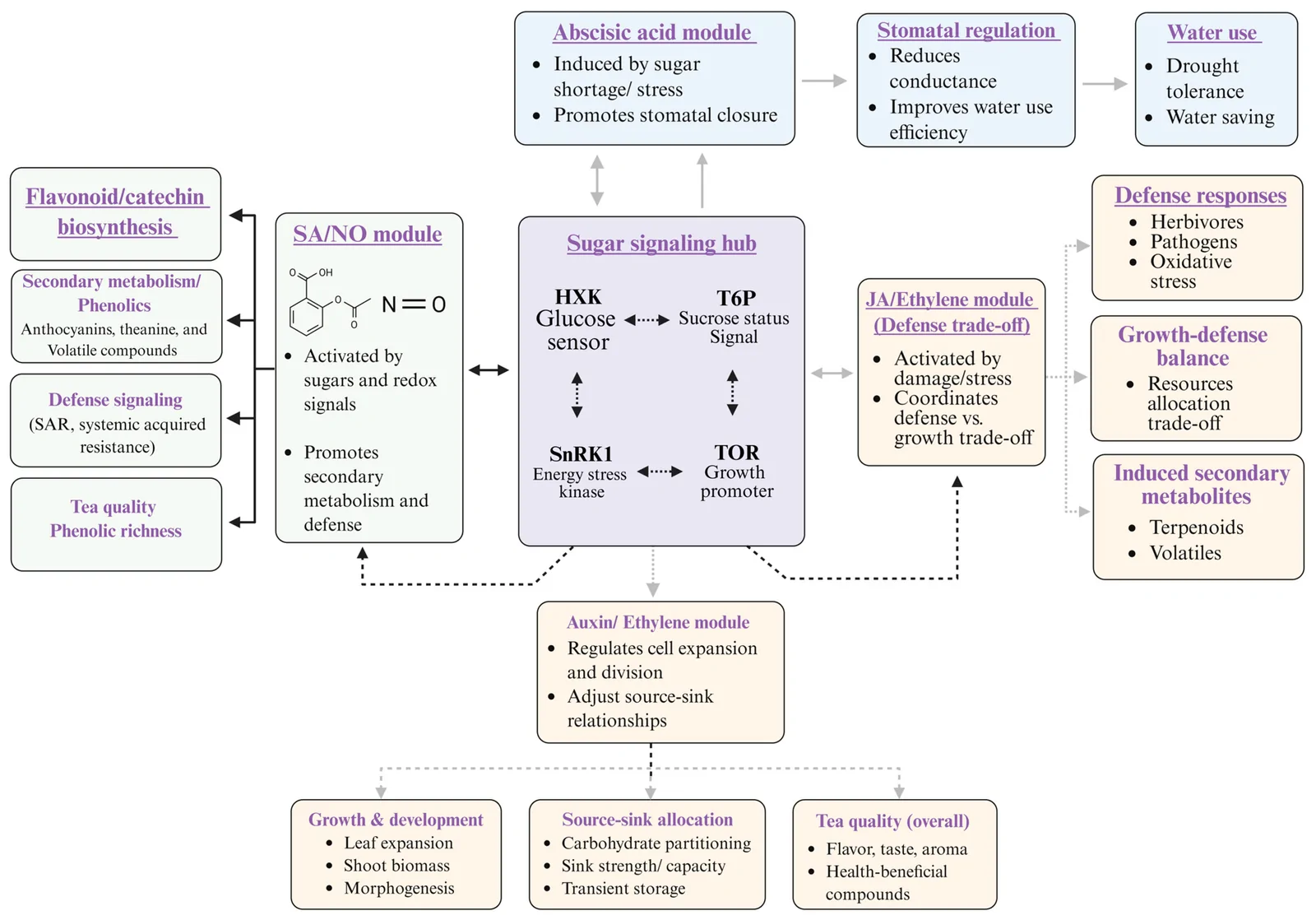
Centralized integration of sugar signaling and multi-hormonal pathways shaping tea quality and adaptation under elevated CO_2_ (eCO_2_). Changes in carbohydrate availability under eCO_2_ are sensed by a core metabolic regulatory hub (HXK, T6P, SnRK1, and TOR). This hub is proposed to interact with major phytohormone modules: an ABA module linked to stomatal regulation and water-use efficiency; a SA/NO module that promotes flavonoid/catechin biosynthesis, phenolic accumulation, and defense signaling (the strongest evidence-based link under eCO_2_ in tea); a JA/ethylene module associated with growth–defense trade-offs and induced secondary metabolites; and an auxin/ethylene module involved in cell expansion, source–sink partitioning, and growth. Together, this multi-layered crosstalk translates upstream nutritional and energy status into coordinated growth, stress responses, and chemical quality traits in *Camellia sinensis*. Solid black arrows indicate relationships directly supported by experimental evidence in tea plants (*Camellia sinensis*) under elevated CO_2_. Solid grey arrows represent conserved mechanisms primarily supported by evidence from other plant species. Dotted arrows denote hypothetical or model-based interactions. Figure created with BioRender.com.

Auxin–ethylene interactions provide another putative connection between carbon availability and growth–defense allocation (Figure 4). In other plant systems, these hormones regulate cell expansion, shoot and root development, senescence, sink formation, and the allocation of resources between growth and defense [61,69]. Because eCO_2_ changes carbohydrate availability and source–sink relationships [70,71,72], auxin and ethylene could modify how additional carbon is distributed among developing shoots, storage tissues, and secondary metabolic pathways. However, no identified tea plants experiment under eCO_2_ has causally linked auxin or ethylene to carbon allocation, shoot development, stress resilience, or fresh-leaf quality [2]. In the present framework, they should therefore be treated as putative modifiers of sink strength and growth–defense balance, not as direct CO_2_ sensors or established mediators of the tea plants’ response.

Overall, the hormonal evidence under eCO_2_ in tea plants is strongly asymmetric. SA–NO regulation of flavonoid biosynthesis represents the clearest directly supported mechanism [23], whereas JA concentrations have been measured directly but vary among experimental contexts [24,26]. Tea plant studies support ABA functions under drought and high light [27,28], but its role in eCO_2_ responses is supported only indirectly. JA–ethylene and auxin–ethylene interactions are hypothetical but derive mainly from tea plant-context or cross-species evidence [25,61,68,69]. Recent synthesis further supports a bidirectional relationship between central metabolic status and phytohormone signaling, with sugar-signaling nodes such as HXK1, T6P, SnRK1, and TOR acting as recurrent points of interaction with hormonal pathways [56]. Moreover, simultaneous coordination among HXK, T6P, SnRK1, TOR, ABA, SA, NO, JA, ethylene, and auxin has not been demonstrated under eCO_2_ in tea plants. Establishing this network will require factorial experiments combining CO_2_ enrichment with defined stress treatments, time-resolved hormone and metabolite profiling, pathway perturbation, and simultaneous measurement of stomatal, defense, growth, and quality traits.

## 5. Stomatal Plasticity Under Elevated CO_2_: Distinguishing Direct CO_2_ Signaling from ABA-Mediated Stress Responses in Tea Plants

Stomata control the principal trade-off between CO_2_ uptake for photosynthesis and transpirational water loss. Their responses to eCO_2_ must be interpreted at two distinct timescales: rapid changes in guard-cell aperture and slower changes in stomatal development [19,73]. Moreover, stomatal conductance (g_s_), aperture, density, and whole-plant water use are not interchangeable measurements. A reduction in g_s_ may reflect altered aperture, stomatal number, pore size, spatial heterogeneity, or combinations of these factors. Consequently, gas-exchange measurements alone cannot identify the underlying regulatory mechanism. The rapid increase in CO_2_ within the leaf can initiate guard-cell CO_2_/HCO_3_^−^ signaling without requiring prior ABA accumulation [19,65]. Recent genetic, biochemical, and gas-exchange analyses in *Arabidopsis* show that CO_2_/HCO_3_^−^ enhances the interaction of MPK12 with the Raf-like kinase HT1 and that CO_2_-dependent stomatal responses require this protein interaction rather than MPK12 catalytic activity [32]. A complementary recent study demonstrated that guard-cell β-carbonic anhydrase 4 directly interacts with the S-type anion channel SLAC1 in a CO_2_-dependent manner, enhancing channel activity and contributing to stomatal CO_2_ responsiveness and kinetics [33]. These findings refine the current model in which CO_2_/HCO_3_^−^ perception is coupled to kinase- and anion-channel-mediated loss of guard-cell turgor and pore closure [19]. Importantly, upstream CO_2_ signaling can remain distinct from ABA perception, although CO_2_- and ABA-responsive pathways may converge on shared downstream components [19,34]. These molecular relationships have not been demonstrated in tea plants under eCO_2_ and should therefore be treated as model-plant mechanisms requiring direct validation in *Camellia sinensis*.

ABA-mediated closure represents a biologically distinct route that becomes particularly important during drought, induced vapor-pressure deficit, salinity, and other conditions that threaten plant water status. Water deficit promotes ABA biosynthesis, transport, or mobilization, followed by ABA perception and the activation of kinase-, Ca^2+^-, ROS-, NO-, and ion-channel-dependent processes that reduce guard-cell turgor [2,28,74]. Tea plant experiments conducted under drought support an ABA-associated role in stress acclimation [28], but these findings do not demonstrate that ABA mediates stomatal responses to eCO_2_ in well-watered tea plants. A decline in g_s_ under CO_2_ enrichment should therefore not be interpreted automatically as evidence of increased ABA concentration or ABA-dependent closure.

The direct CO_2_ and ABA-mediated routes may converge at downstream components, including Ca^2+^ dynamics, ROS and NO signaling, OST1-related regulation, and anion-channel activation [28,34,74]. This convergence explains how CO_2_ enrichment and drought can produce similar stomatal outcomes while originating from different upstream signals. Shared downstream effectors do not, however, make ABA the guard-cell CO_2_ sensor. Under combined eCO_2_ and water deficit, both routes may operate simultaneously or interact, but their relative contributions can be resolved only through factorial experiments that manipulate CO_2_ and water status independently while measuring guard-cell ABA, aperture kinetics, and pathway activity.

Tea plant studies under eCO_2_ mostly document physiological responses rather than the associated molecular mechanisms. Controlled eCO_2_ studies have reported increased photosynthetic assimilation and reduced g_s_, but they generally have not quantified stomatal aperture kinetics, guard-cell ABA, CO_2_/HCO_3_^−^-signaling components, or the relative contributions of CO_2_-specific and ABA-dependent pathways [2,17]. Tea plants’ gas exchange also exhibits diurnal and spatial heterogeneity, complicating interpretation of calculated intercellular CO_2_ and stomatal limitation [75]. Direct epidermal imaging and aperture measurements are therefore required before a decrease in g_s_ can be assigned specifically to rapid pore closure. The water-use consequences also require precise terminology. An increase in A/g_s_ supports greater intrinsic water-use efficiency, whereas A/E represents instantaneous leaf-level water-use efficiency [21,26,76]. Neither measurement alone demonstrates lower daily, whole-plant, canopy, or plantation water consumption. Changes in leaf area, canopy temperature, boundary-layer conductance, soil evaporation, and treatment duration may alter the final water balance even when leaf-level efficiency increases.

**Figure 5:**
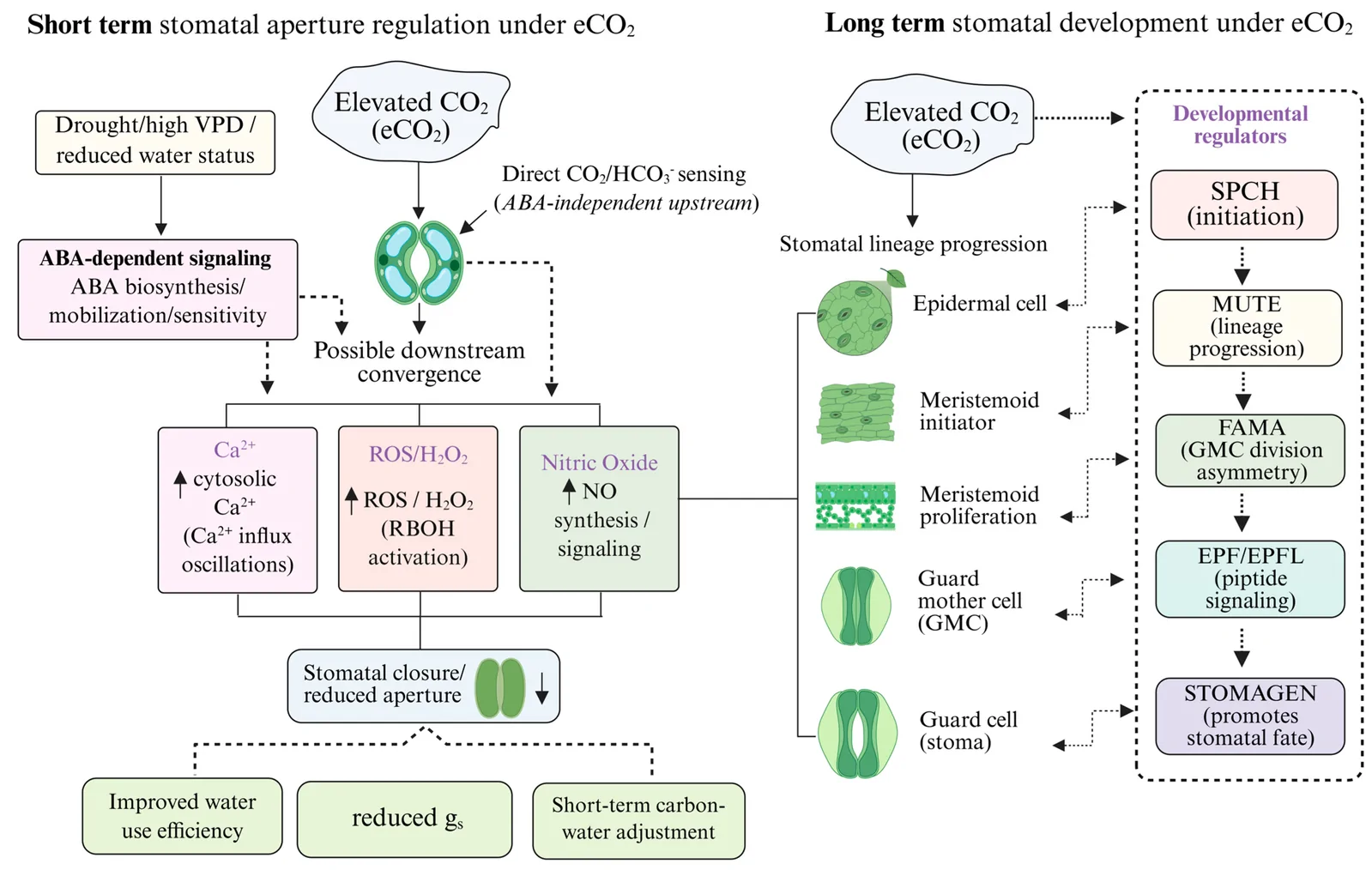
Distinct rapid and developmental routes controlling stomatal responses under elevated CO_2_ (eCO_2_). The left panel shows short-term stomatal aperture regulation. Elevated CO_2_ triggers an ABA-independent direct CO_2_/HCO_3_^−^ sensing pathway in guard cells that converges with secondary messengers (Ca^2+^, ROS/H_2_O_2_, and nitric oxide) to promote stomatal closure and reduced aperture. An ABA-dependent pathway, activated by drought or reduced water status, can also contribute and may converge downstream. These rapid responses improve water-use efficiency and lower stomatal conductance (g_s_). The right panel illustrates long-term stomatal development under eCO_2_. Elevated CO_2_ influences stomatal lineage progression through developmental regulators (SPCH, MUTE, FAMA, EPF/EPFL peptides, and STOMAGEN). Together, these rapid and developmental mechanisms allow plants to adjust both instantaneous aperture and long-term stomatal density in response to elevated CO_2_. Solid black arrows indicate relationships directly supported by experimental evidence in tea plants (*Camellia sinensis*) under elevated CO_2_. Solid grey arrows represent conserved mechanisms primarily supported by evidence from other plant species. Dotted arrows denote hypothetical or model-based interactions. Figure created with BioRender.com.

Stomatal responses sustained over days or weeks must also be distinguished from rapid aperture regulation. In *Arabidopsis*, SPEECHLESS (SPCH), MUTE, and FAMA regulate successive stages of stomatal-lineage initiation, meristemoid differentiation, and guard-cell formation, respectively [73,77,78,79,80]. EPF/EPFL peptide signaling and STOMAGEN further regulate stomatal initiation and spacing [81]. Recent evidence shows that SPCH and FAMA can also function during the late stomatal lineage to regulate guard-cell division and expansion [82], while ABA can influence stomatal proliferation and patterning through the EPF–SPCH pathway in *Arabidopsis* [83,84]. However, corresponding regulation of these developmental pathways under eCO_2_ has not been demonstrated in tea plants. These mechanisms should therefore be treated as developmental hypotheses requiring tea plant-specific validation. Reliable attribution will require experiments combining time-resolved gas exchange with direct measurements of pore aperture, stomatal density, stomatal index, guard-cell size, and spatial patterning, together with ABA quantification and expression or activity analyses of candidate CO_2_- and ABA-signaling components (Figure 5). Such approaches would distinguish rapid CO_2_-specific closure from stress-induced ABA regulation and longer-term developmental acclimation.

## 6. Reporting CO_2_ Exposure: Distinguishing Atmospheric Enrichment from Biological Carbon Gain

Tea plant eCO_2_ experiments are commonly described by a target CO_2_ concentration, although treatment duration, daily enrichment period, concentration stability, and actual canopy-level exposure can differ substantially among studies [2,17,22]. Reporting concentration alone therefore provides an incomplete description of the imposed treatment. A complementary descriptor is the cumulative enrichment above a simultaneously measured ambient reference:

$$E_{\Delta CO_{2}}=\int_{0}^{T}[C_{treatment}\left(t\right)-C_{reference}\left(t\right)]dt$$

where $C_{treatment}\left(t\right)$ and $C_{reference}\left(t\right)$ are synchronized canopy-level CO_2_ concentrations over the defined enrichment interval. When concentration is expressed as μmol mol^−1^ and time in hours, the resulting exposure can be reported as Δppm·h. The calculation should be based on logged CO_2_ concentrations rather than nominal chamber set points, with the sampling interval, daily enrichment schedule, photoperiod, and interruptions in CO_2_ delivery reported explicitly.

Importantly, Δppm·h describes the atmospheric treatment, not the amount of carbon assimilated by the plant. Realized carbon gain depends on irradiance, temperature, vapor-pressure deficit, stomatal conductance, photosynthetic capacity, nitrogen availability, sink demand, developmental stage, and genotype [21,40,51,71]. For assimilation-dependent responses, cumulative net carbon gain can therefore be considered separately:

$$G_{C}=\int_{0}^{T}A_{net}\left(t\right)dt$$

where $A_{net}\left(t\right)$ is the measured net photosynthetic rate over the defined interval. $E_{\Delta CO_{2}}$ and $G_{C}$ describe different quantities: the former characterizes atmospheric exposure, whereas the latter estimates realized leaf-level carbon assimilation. Neither measurement alone establishes how newly fixed carbon is subsequently partitioned among young shoots, roots, storage pools, or quality-related metabolites.

The biological relevance of cumulative exposure should therefore be treated as a testable hypothesis rather than an established dose metric. Experiments could compare continuous, pulsed, or shorter high-concentration enrichment programs that produce similar cumulative exposure while differing in concentration history. Comparable responses would suggest that cumulative exposure captures useful treatment information, whereas divergent responses would indicate that peak concentration, timing, recovery intervals, or physiological kinetics contain information not represented by Δppm·h. This distinction is especially important for rapid stomatal responses, which should be evaluated against instantaneous CO_2_ concentration and response kinetics rather than cumulative exposure alone.

Nitrogen supply, water status, sink strength, cultivar, developmental stage, and harvest regime should likewise be treated as biological modifiers [13,17,43]. Consequently, no universal threshold, responsive, or saturation zones can presently be assigned to Δppm·h in tea plants. Its usefulness should instead be evaluated alongside instantaneous or mean CO_2_ concentration, temporal exposure pattern, integrated carbon assimilation, environmental covariates, and direct indicators of carbon allocation such as biomass partitioning or ^13^CO_2_ tracing. Because daily enrichment duration may differ among experiments, Δppm·h should be reported together with the number of enrichment hours per photoperiod rather than normalized in a way that obscures the actual exposure schedule. Until such validation is available, Δppm·h is best regarded as a standardized treatment-reporting descriptor and hypothesis-generating variable, rather than a validated biological dose, optimization target, or predictor of tea quality.

## 7. Conclusions and Future Perspectives

Elevated CO_2_ alters tea-plant physiology by affecting carbon assimilation, source–sink relations, carbon–nitrogen coordination, and carbohydrate status, while its effects on hormone-associated signaling remain less completely resolved. Current evidence most clearly supports changes in photosynthetic performance, carbohydrate accumulation, and several quality-related metabolites. Among the proposed hormonal mechanisms, the salicylic acid–nitric oxide pathway has the strongest direct support in tea plants, particularly in flavonoid biosynthesis. However, altered metabolite concentrations or increased intrinsic water-use efficiency should not automatically be interpreted as improved stress resilience or product quality.

The proposed links between sugar signaling and phytohormone regulation remain largely mechanistic hypotheses in tea plants. HXK, T6P, SnRK1, and TOR are putative regulators of carbon-status sensing and metabolic allocation, but their coordinated operation under eCO_2_ has not been demonstrated directly in *Camellia sinensis*. Likewise, ABA should not be treated as the universal mediator of CO_2_-induced stomatal responses; jasmonate responses appear context-dependent, and ethylene- and auxin-associated pathways require tea plant-specific validation.

Future work should prioritize factorial, time-resolved experiments integrating eCO_2_ with nitrogen status, source–sink manipulation, and environmental stress, together with gas exchange, stomatal kinetics, sugar and hormone profiling, kinase activity, stable-isotope tracing, and multi-omics. Cumulative enrichment (Δppm·h) should presently be treated only as an experimental exposure descriptor and tested against alternative measures of CO_2_ exposure and realized carbon gain. Ultimately, precision CO_2_ management in tea plants will require clear separation of fresh-leaf composition, processed-tea chemistry, sensory quality, and stress resilience as distinct outcomes.

## Figures and Tables

**Table 1:** Evidence context and causal support for principal relationships linking eCO_2_, carbon status, phytohormone signaling, stomatal regulation, and quality-related metabolism in tea plants.

Relationship Evaluated	Species/System	Experimental Context	Evidence Type	Causal Support	Maximum Inference Justified	References
eCO_2_ → increased photosynthesis, carbohydrates, and altered C:N/metabolite profiles	*Camellia sinensis*	Controlled eCO_2_; 800 μmol mol^−1^ for 24 d	Gas exchange, biochemical profiling, gene expression	Direct treatment response; not pathway mediation	eCO_2_ alters photosynthetic performance, carbohydrate pools, C:N balance, and several fresh-leaf metabolites; this does not by itself demonstrate improved processed-tea quality	[17]
eCO_2_ → SA → NO → PAL/flavonoid response	*Camellia sinensis*	Ambient and eCO_2_ with SA/NO pharmacological perturbation	Hormone and flavonoid measurements; pathway inhibition/scavenging	Pharmacological perturbation-supported causal ordering	SA contributes upstream of part of the NO–PAL-associated flavonoid response under eCO_2_; this is the strongest tea plant-specific causal relationship identified here	[23]
eCO_2_ → altered caffeine/JA-associated defense against Colletotrichum gloeosporioides	*Camellia sinensis* ‘Longjing 43’	380 vs. 800 μmol mol^−1^ CO_2_ for 2 weeks followed by fungal challenge; caffeine/MeJA treatments	Metabolites, disease phenotype, LOX activity/expression, exogenous perturbation	Context-specific perturbation evidence	eCO_2_ can modify caffeine- and JA-associated defense under this pathogen system; the response should not be generalized to unstressed tea plants or all stressors	[24]
eCO_2_ → changes in soluble nutrients, JA, SA and aphid abundance	*Camellia sinensis* + Toxoptera aurantii	eCO_2_ with herbivore interaction	Gas exchange, metabolites, hormones, transcripts, insect population	Direct treatment association; no demonstrated hormonal mediation	eCO_2_ alters foliar chemistry and hormone abundance in this herbivore context, but measured JA/SA changes do not establish that either hormone mediates the aphid response	[26]
ABA → anthocyanin accumulation under high light	*Camellia sinensis*	High-light treatment with ABA-related manipulation	Hormone analysis, expression and pharmacological evidence	Tea plant-specific perturbation evidence outside eCO_2_	ABA participates in high-light-induced anthocyanin regulation in tea plants; this does not demonstrate ABA mediation of eCO_2_ responses	[27]
Ethylene signaling → catechin/antioxidant responses	*Camellia sinensis*	Ethylene-related treatment outside eCO_2_	Hormone manipulation and biochemical analysis	Tea plant-context physiological evidence	Ethylene can modify catechin-related metabolism in tea plants, but an eCO_2_–ethylene causal pathway has not been demonstrated	[25]
Tea plant nitrogen deprivation/resupply → coordinated root N metabolism and secondary-metabolite responses	*Camellia sinensis*	N deprivation followed by resupply	Metabolomics + RNA-seq	Direct N-treatment response; no eCO_2_ evidence	Tea plants possess dynamic N-acquisition, assimilation, and remobilization capacity that could condition eCO_2_ responses, but eCO_2_ regulation of these processes remains untested	[29]
NPF and AMT2 transporter families → candidate N uptake control points	*Camellia sinensis*	Genomic/expression analyses under N-related conditions	Genome-wide identification, expression profiling	Candidate molecular evidence	NPF and AMT2 members are putative determinants of tea plant N acquisition, but no evidence yet shows that eCO_2_ regulates these transporters	[30,31]
MPK12–HT1 interaction → stomatal CO_2_ signaling	*Arabidopsis thaliana*	CO_2_/HCO_3_^−^-responsive guard-cell signaling	Genetic, biochemical and structural analyses	Strong mechanistic evidence in a model plant	MPK12–HT1 interaction is a well-supported component of stomatal CO_2_ signaling in Arabidopsis; conservation in tea plants remains untested	[32]
βCA4–SLAC1 interaction → CO_2_-responsive anion-channel regulation	*Arabidopsis thaliana*	Guard-cell CO_2_ signaling	Genetic, biochemical and electrophysiological analyses	Strong mechanistic evidence in a model plant	βCA4 can directly regulate SLAC1-associated CO_2_ responsiveness; whether this mechanism operates in tea plants remains unknown	[33]
HXK as a glucose-status sensor and regulator	Primarily *Arabidopsis thaliana*/model plants	Sugar-signaling studies, not tea plant eCO_2_	Genetic and biochemical evidence	Strong model-plant mechanistic evidence	HXK is a putative carbon-status node, but eCO_2_-dependent HXK signaling has not been demonstrated in tea plants	[18,35]
T6P–SnRK1 regulation and integration with energy-status signaling	Primarily *Arabidopsis. thaliana*/model plants	Sugar/energy signaling, not tea plant eCO_2_	Biochemical and genetic evidence; recent synthesis	Mechanistic evidence outside tea plant	T6P–SnRK1 interaction provides a putative route linking sucrose status with energy regulation, but its operation under eCO_2_ in tea plants remains untested; it should not be portrayed as a simple linear HXK → T6P → SnRK1 → TOR pathway	[36,38]
Integrated HXK–T6P–SnRK1–TOR control of tea plant responses to eCO_2_	*Camellia sinensis*	Not directly tested	Conceptual integration of tea plant physiology with model-plant mechanisms	Hypothesis	The coordinated network is a testable mechanistic hypothesis, not an established tea plant eCO_2_ pathway	No direct tea plant experiment

**Notes:** Experimental context and causal support are evaluated independently. “Direct treatment response” indicates that an endpoint changed following the imposed treatment but does not establish mediation by the measured signaling component. “Pharmacological perturbation-supported” indicates that pathway interference altered the response and therefore provides stronger evidence for causal ordering, although pharmacological specificity should still be considered. Model-plant mechanisms are not assumed to be conserved in *Camellia sinensis*. ABA, abscisic acid; βCA4, beta-carbonic anhydrase 4; C:N, carbon-to-nitrogen ratio; HXK, hexokinase; JA, jasmonic acid; LOX, lipoxygenase; NO, nitric oxide; NPF, NRT1/PTR FAMILY; PAL, phenylalanine ammonia-lyase; SA, salicylic acid; SLAC1, SLOW ANION CHANNEL-ASSOCIATED 1; SnRK1, SNF1-related protein kinase 1; T6P, trehalose 6-phosphate; TOR, target of rapamycin.

## Data Availability

No new data were created or analyzed in this study. Data sharing is not applicable to this article.

## Supplementary Materials

The following supporting information can be downloaded at: https://www.mdpi.com/article/10.3390/biom16101447/s1, Table S1: Study-level evidence inventory for relationships linking elevated CO_2_ with carbon status, phytohormone signaling, stomatal regulation, nitrogen coordination, stress responses, and quality-related metabolism in tea plants. References cited in Table S1 are included in the main manuscript reference list.
