# Supplementary material for: Phytohormone Crosstalk Under Elevated CO2: Linking Sugar Signaling, Stress Resilience, and Metabolic Quality in Tea
Source: Biomolecules. 2026 Oct 4;16(10):1447. doi: 10.3390/biom16101447 (PMC13702587; doi:10.3390/biom16101447)
Supplement: Supplementary file 1 [file biomolecules-16-01447-s001.zip › biomolecules-4536976-supplementary.pdf]

Supplementary Materials: Table S1: Study-level evidence inventory for relationships linking elevated CO_2 with carbon status, phytohormone signaling, stomatal regulation, nitrogen coordination, stress responses, and quality-related metabolism in tea plants. References cited in Table S1 are included in the main manuscript reference list.

No.	Relationship or study focus	Species/material	Experimental context and key conditions	Measurements/perturbation	Evidence context	Causal support	Maximum inference justified	Reference
1	eCO_2 → photosynthesis, carbohydrate accumulation, C:N balance and quality-related metabolites	*Camellia sinensis* cv. Longjing 43; 2-year-old seedlings	Controlled-environment chambers; 380 vs 800 μmol mol⁻¹ CO_2; 24 d; four randomized blocks	Gas exchange, respiration, biomass, soluble sugars, starch, C and N, amino acids, catechins, caffeine, qPCR	Direct tea evidence under eCO_2	Direct treatment-response evidence; no pathway perturbation	eCO_2 alters photosynthetic performance, carbohydrate pools, C:N balance and multiple fresh-leaf metabolites. These responses do not establish signaling mediation or improved processed-tea quality	[25]
2	eCO_2 → SA → NO → PAL-associated flavonoid accumulation	*Camellia sinensis* cv. Longjing 43; 2-year-old seedlings	Controlled chambers; 400, 550 and 800 μmol mol⁻¹ CO_2; subsequent mechanistic experiments at elevated CO_2	SA and NO quantification; flavonoids; PAL activity; SA biosynthesis inhibition; NO scavenging; exogenous SA and NO donor	Direct tea evidence under eCO_2	Pharmacological perturbation-supported causal ordering	SA contributes upstream of part of the NO–PAL–flavonoid response under eCO_2. This is the strongest tea-specific causal relationship identified in the review, but pharmacological specificity remains a limitation	[26]
3	eCO_2 → reduced caffeine/JA-associated defense against Colletotrichum gloeosporioides	*Camellia sinensis* cv. Longjing 43; 2-year-old seedlings	Controlled chambers; 380 vs 800 μmol mol⁻¹ CO_2 for 14 d; subsequent fungal inoculation	Caffeine, JA, LOX activity/expression, pathogen symptoms/actin, caffeine and MeJA application	Direct tea eCO_2 × pathogen evidence	Treatment response plus pharmacological perturbation	eCO_2 modified caffeine- and JA-associated defense in this specific pathogen context. The result cannot be generalized to unstressed plants or all biotic stresses	[28]
4	eCO_2 → altered photosynthesis, metabolites, JA/SA and tea-aphid population	*Camellia sinensis* cv. Longjing Changye; 1-year-old seedlings + Toxoptera aurantii	Open-top chambers; approximately 770 μmol mol⁻¹ eCO_2; 60 d	Gas exchange, biomass, soluble nutrients, theanine, polyphenols, caffeine, JA, SA, transcript abundance, aphid population	Direct tea eCO_2 × herbivore evidence	Treatment association; no hormonal pathway perturbation	eCO_2 altered tea chemistry, JA/SA abundance and aphid population in this herbivore system, but the measured hormone changes do not establish hormonal mediation of the aphid response	[27]
5	ABA involvement in high-light-induced anthocyanin accumulation	*Camellia sinensis* cv. Zifuxing 1 and additional purple/green cultivars	Seasonal observations; shade treatment; full-sun ABA and fluridone treatments	ABA, anthocyanins, other hormones, pigments, antioxidant activity, RNA-seq, ABA application and ABA-biosynthesis inhibition	Tea-specific evidence outside eCO_2	Correlative evidence plus pharmacological perturbation	ABA contributes to high-light-associated anthocyanin regulation in tea plants; this does not demonstrate that ABA mediates the eCO_2 response	[29]
6	ABA-associated response to drought	*Camellia sinensis*	Drought stress with exogenous ABA	Proteomic and physiological responses	Tea-specific evidence outside eCO_2	Treatment-response evidence	ABA influences tea-plant drought responses and carbon-related metabolism, but its role in eCO_2-induced stomatal responses remains untested	[30]

No.	Relationship or study focus	Species/material	Experimental context and key conditions	Measurements/perturbation	Evidence context	Causal support	Maximum inference justified	Reference
7	Ethylene signaling → catechin and antioxidant-related responses	*Camellia sinensis*	Ethylene-signaling manipulation outside the eCO_2 context	Catechin composition and antioxidant-related measurements	Tea-specific evidence outside eCO_2	Physiological/biochemical treatment evidence	Ethylene signaling can modify catechin-related metabolism in tea plants, but no causal eCO_2–ethylene pathway has been demonstrated	[31]
8	N deprivation/resupply → root N metabolism and quality-related metabolites	*Camellia sinensis* roots	N deprivation followed by N resupply	Untargeted metabolomics, RNA-seq, qRT-PCR; amino acids, polyphenols, caffeine; N-related genes/transporters	Tea-specific evidence outside eCO_2	Direct N-treatment response	Tea plants possess dynamic N assimilation/remobilization responses that could condition eCO_2 effects; the study does not demonstrate regulation of these processes by eCO_2	[32]
9	NPF transporter family → candidate nitrate-uptake/regulatory capacity	*Camellia sinensis*	Genome-wide characterization and expression analyses; no eCO_2 treatment	Identification of 109 CsNPF genes, phylogeny, promoter and expression analyses	Tea-specific evidence outside eCO_2	Genomic/expression evidence	CsNPFs identify potential nitrate-transport control points in tea, but eCO_2 regulation or causal involvement has not been established	[33]
10	AMT2 transporters → ammonium acquisition differences among tea cultivars	*Camellia sinensis* cultivars Fudingdabaicha, Huangdan and Maoxie	NH_4^+ uptake comparisons among cultivars; no eCO_2 treatment	Genome-wide AMT2 identification, root expression, NH_4^+ uptake kinetics	Tea-specific evidence outside eCO_2	Physiological and molecular association	CsAMT2 members are plausible determinants of tea ammonium acquisition, but there is no evidence that eCO_2 acts through these transporters	[34]
11	MPK12–HT1 interaction → guard-cell CO_2/HCO_3^- sensing	*Arabidopsis thaliana;* complementary protein-interaction assays	Low/high CO_2 and HCO_3^- manipulations; not tea	Mutants, gas exchange, biochemical interaction assays, protein-complex analyses, structural modelling	Model-plant mechanistic evidence	Genetic + biochemical mechanistic evidence	CO_2/HCO_3^--dependent MPK12–HT1 interaction is strongly supported in Arabidopsis; conservation in tea remains untested	[35]
12	βCA4–SLAC1 interaction → CO_2-dependent anion-channel regulation	*Arabidopsis thaliana;*	Guard-cell CO_2-response experiments; near-ambient/elevated CO_2 contexts	Protein interaction, mutants, electrophysiology/channel activity, stomatal kinetics, water-use efficiency	Model-plant mechanistic evidence	Genetic + biochemical + electrophysiological evidence	βCA4 can interact directly with SLAC1 and contribute to CO_2 responsiveness in Arabidopsis; this mechanism has not been demonstrated in tea	[36]
13	ABA-independent upstream CO_2 signaling and downstream convergence with ABA	*Arabidopsis thaliana;*	Rapid CO_2 elevation in ABA biosynthesis and receptor mutants	Gas exchange, ABA reporter imaging, mutants, kinase assays, patch clamp/anionic-channel measurements	Model-plant mechanistic evidence	Genetic + physiological + biochemical evidence	Rapid stomatal CO_2 responses can proceed without an increase in guard-cell ABA, although basal ABA signaling can modulate response kinetics and pathways converge downstream	[37]
14	HXK1 as a glucose-status sensor regulating	*Arabidopsis thaliana;* and model systems	Sugar-signaling experiments; no tea eCO_2 treatment	Genetic and biochemical analysis of HXK-dependent glucose signaling	Model-plant mechanistic evidence	Genetic/biochemical evidence	HXK is a plausible glucose-status regulator linking carbon availability with gene expression, but eCO_2-	[38]

No.	Relationship or study focus	Species/material	Experimental context and key conditions	Measurements/perturbation	Evidence context	Causal support	Maximum inference justified	Reference
	growth/photosynthetic gene expression						dependent HXK signaling has not been demonstrated in tea plants	
15	T6P inhibition of SnRK1	Model plants / plant biochemical systems	Manipulation of T6P and SnRK1 activity; no tea eCO_2 treatment	Enzyme activity and metabolic analyses	Cross-species mechanistic evidence	Biochemical evidence	T6P can inhibit SnRK1 and link sucrose/carbon status with metabolic regulation; operation of this mechanism under eCO_2 in tea remains untested	[39]
16	SnRK1–TOR coordination of cellular energy/growth status	Model plants and broader plant literature	Carbon/energy-signaling contexts; no direct tea eCO_2 experiment	Genetic, biochemical and integrative mechanistic evidence	Cross-species mechanistic evidence	Mechanistic support outside tea	SnRK1 and TOR participate in interacting energy- and growth-regulatory networks, but they should not be represented as a single validated linear pathway under eCO_2 in tea	[40,41]
17	Integrated HXK–T6P–SnRK1–TOR signaling under eCO_2	*Camellia sinensis*	No direct experiment identified	Proposed integration of eCO_2-induced carbohydrate change with model-plant sugar/energy signaling	Untested directly in tea	Hypothesis	The integrated network is a testable hypothesis; simultaneous activation, temporal ordering and causal mediation have not been demonstrated under eCO_2 in tea plants	No direct tea study
18	JA–ethylene coordination of eCO_2-induced defense/volatile metabolism	*Camellia sinensis*	No direct factorial/causal tea experiment identified	Tea JA observations combined with tea-context/cross-species ethylene evidence	Partly tea-context; partly cross-species	Hypothesis in the eCO_2 context	JA responses to eCO_2 are context dependent; a causal JA–ethylene module connecting eCO_2 with defense or aroma metabolism remains unvalidated in tea	[26-28,31]
19	Auxin–ethylene regulation of eCO_2-dependent source–sink allocation	*Camellia sinensis*	No direct experiment identified	Conceptual extrapolation from cross-species developmental/hormonal evidence	Cross-species evidence / untested tea relationship	Hypothesis	Auxin and ethylene are plausible modifiers of sink development and growth–defense allocation, but they are not established mediators of eCO_2 responses in tea	Relevant cross-species literature
20	eCO_2 → processed-tea sensory quality	*Camellia sinensis*	Direct eCO_2 studies mainly measured fresh-leaf physiology and chemical composition	Metabolite concentrations; limited/no integrated processing + sensory validation	Evidence gap	No direct causal support identified	Changes in catechins, theanine, caffeine or volatiles should not automatically be described as improved tea quality without processed-tea chemistry and sensory assessment	Evidence gap identified in this review

Notes: Evidence context and cause and support are evaluated separately. "Direct tea evidence under eCO_2" indicates that the relationship was examined in *Camellia sinensis* exposed to eCO_2, whereas "tea-specific evidence outside eCO_2" identifies mechanisms demonstrated in tea plants under another treatment context. "Model-plant/cross-species evidence" denotes mechanistic evidence obtained outside *Camellia sinensis* and therefore does not establish conservation in tea. A "direct treatment response" indicates that an

endpoint changed following the imposed treatment but does not establish mediation by the measured signaling component. “Pharmacological perturbation-supported” indicates that pathway interference altered the response and therefore provides stronger evidence for causal ordering, while remaining subject to pharmacological specificity. “Genetic/biochemical mechanistic evidence” denotes stronger causal support in the species studied but is not automatically transferable to tea. Rows described as “hypothesis” identify relationships for which no direct tea-plant validation under elevated CO_2 was identified. eCO_2, elevated carbon dioxide; ABA, abscisic acid; βCA4, beta-carbonic anhydrase 4; C:N, carbon-to-nitrogen ratio; HCO_3^-, bicarbonate; HXK, hexokinase; JA, jasmonic acid; LOX, lipoxygenase; MPK12, mitogen-activated protein kinase 12; NPF, NRT1/PTR FAMILY; NO, nitric oxide; PAL, phenylalanine ammonia-lyase; SA, salicylic acid; SLAC1, SLOW ANION CHANNEL-ASSOCIATED 1; SnRK1, SNF1-related protein kinase 1; T6P, trehalose 6-phosphate; TOR, target of rapamycin.
